# Transcatheter closure of a giant and tortuous right coronary artery-to-right atrium fistula in a child: a case report

**DOI:** 10.3389/fped.2026.1895707

**Published:** 2026-09-18

**Authors:** Hana Christiani Sembiring, Putri Primawardani, Mulyadi M. Djer

**Affiliations:** 1 Pediatrics Subspecialist Training Program, Department of Child Health, Medical Faculty, University of Indonesia, Cipto Mangunkusumo Hospital, Jakarta, Indonesia; 2 Pediatric Cardiology Division, Department of Child Health, Medical Faculty, University of Indonesia, Cipto Mangunkusumo Hospital, Jakarta, Indonesia

**Keywords:** Amplatzer® Vascular Plug II, coronary artery fistula, pediatric, right coronary artery, transcatheter closure (TCC)

## Abstract

Coronary artery fistula (CAF) is a rare congenital coronary anomaly characterized by an abnormal communication between a coronary artery and a cardiac chamber or vascular structure. Large fistulas may produce significant left-to-right shunting and progressive cardiac volume overload. We report a successful transcatheter closure of a giant and tortuous right coronary artery (RCA) to right atrium (RA) fistula in a 4-year-2-month-old girl using an Amplatzer® Vascular Plug II (AVP II) device. Echocardiography demonstrated a markedly dilated RCA fistula draining into the RA, with mild dilatation of the right atrium and right ventricle. Cardiac catheterization confirmed a giant and tortuous fistulous tract with aneurysmal dilatation. During the first attempt, the unreleased device became unstable and displaced but was successfully recaptured. The fistula was subsequently closed using an arteriovenous loop technique, without lasting clinical sequelae. Final angiography demonstrated complete occlusion without residual shunt or coronary compromise. Follow-up echocardiography showed the device to be well positioned without residual flow. This case highlights the feasibility of transcatheter closure in selected pediatric patients with complex coronary artery fistula anatomy.

## Introduction

Coronary artery fistulas (CAFs) are rare congenital coronary anomalies characterized by abnormal communication between a coronary artery and a cardiac chamber or vascular structure, thereby bypassing the normal myocardial capillary network. The reported prevalence ranges from approximately 0.2% to 1.2%, with increasing detection rates because of advances in echocardiographic and angiographic imaging modalities (1, 2).

Most CAFs originate from the right coronary artery and commonly drain into right-sided cardiac chambers because of lower-pressure receiving systems. The clinical presentation varies depending on the size and hemodynamic significance of the fistula. Small fistulas may remain asymptomatic, whereas large fistulas can result in significant left-to-right shunting, coronary steal phenomenon, chamber dilatation, arrhythmia, myocardial ischemia, and heart failure (1, 3).

Echocardiography remains the first-line imaging modality for detecting CAFs, whereas cardiac catheterization is essential for detailed anatomical assessment and procedural planning. In appropriately selected patients, transcatheter closure has become the preferred treatment because it preserves native coronary circulation while avoiding sternotomy. However, giant aneurysmal and markedly tortuous CAFs remain technically demanding, and optimal procedural strategies for device stabilization and redeployment have been only sparsely described (3, 4).

We report the successful transcatheter closure of a giant tortuous right coronary artery-to-right atrium fistula with aneurysmal dilatation in a pediatric patient using an AVP II. Beyond successful closure, this case highlights practical technical strategies to facilitate transcatheter management of complex coronary artery fistulas.

## Case presentation

A 4-year-2-month-old girl was referred to pediatric cardiology outpatient clinic at Cipto Mangunkusumo National Central General Hospital for evaluation of suspected congenital heart disease. The patient had a history of recurrent pulmonary infections since the age of 6 months and accompanied by poor weight gain. At the age of 3 years, she was hospitalized due to bronchopneumonia and a cardiac murmur was identified for the first time by paediatrician. The parents declined further echocardiographic examination for financial constraints. During the three months before presentation, the parents observed the patient reduced exercise tolerance and the child appearing smaller than her peers. Consequently, they brought her back for further evaluation and management. Figure 1 summarized the patient's clinical course from symptom onset to follow up after intervention.

**Figure 1:**
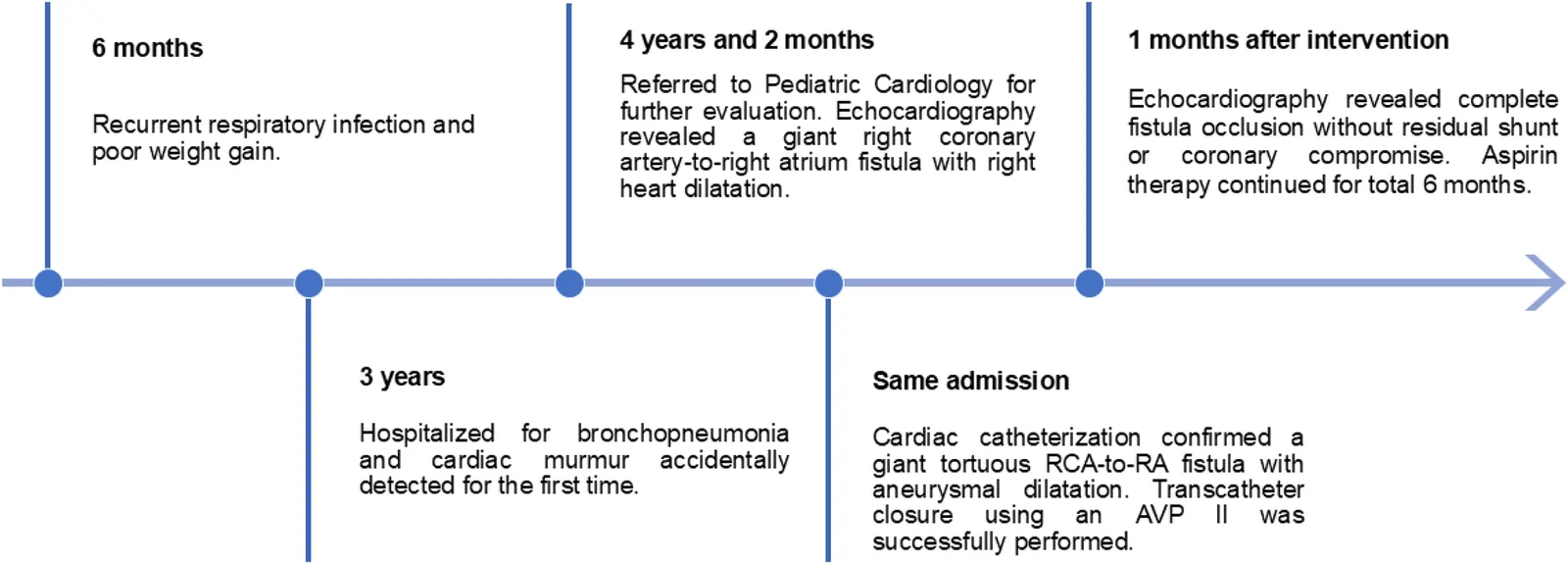
CARE timeline summarizing the patient clinical course.

On physical examination, the patient was fully alert and hemodynamically stable. The body weight was 11.4 kg and body height was 93 cm. Anthropometric assessment indicated that the patient was underweight (weight-for-age = −3 < z-score < −2 SD), stunted (height-for-age = −3 < z-score < −2 SD), and well-nourished (weight-for-height = −2 < z-score <−1 SD). Cardiovascular examination revealed normal first and second heart sounds with a continuous murmur at third to fourth right intercostal space along right parasternal border. No gallop rhythm was noted. Pulmonary examination demonstrated no wheezing or crackles.

Electrocardiography showed sinus tachycardia, normal cardiac axis, no evidence of right or left chamber enlargement and without sign of myocardial ischemia. Preprocedural transthoracic echocardiography demonstrated mildly dilated right atrium and ventricle, mild tricuspid regurgitation, structurally normal cardiac valves with estimated Qp/Qs ratio of 2.5. Apical four chambers and parasternal long axis view identified dilatation of right coronary artery draining into an aneurysmal fistulous sac at right atrium (Figures 2A,B). The patient was treated with furosemide 5 mg twice daily and captopril 3.125 mg three times daily for 3 months. Transcatheter closure of the fistula was subsequently planned.

**Figure 2:**
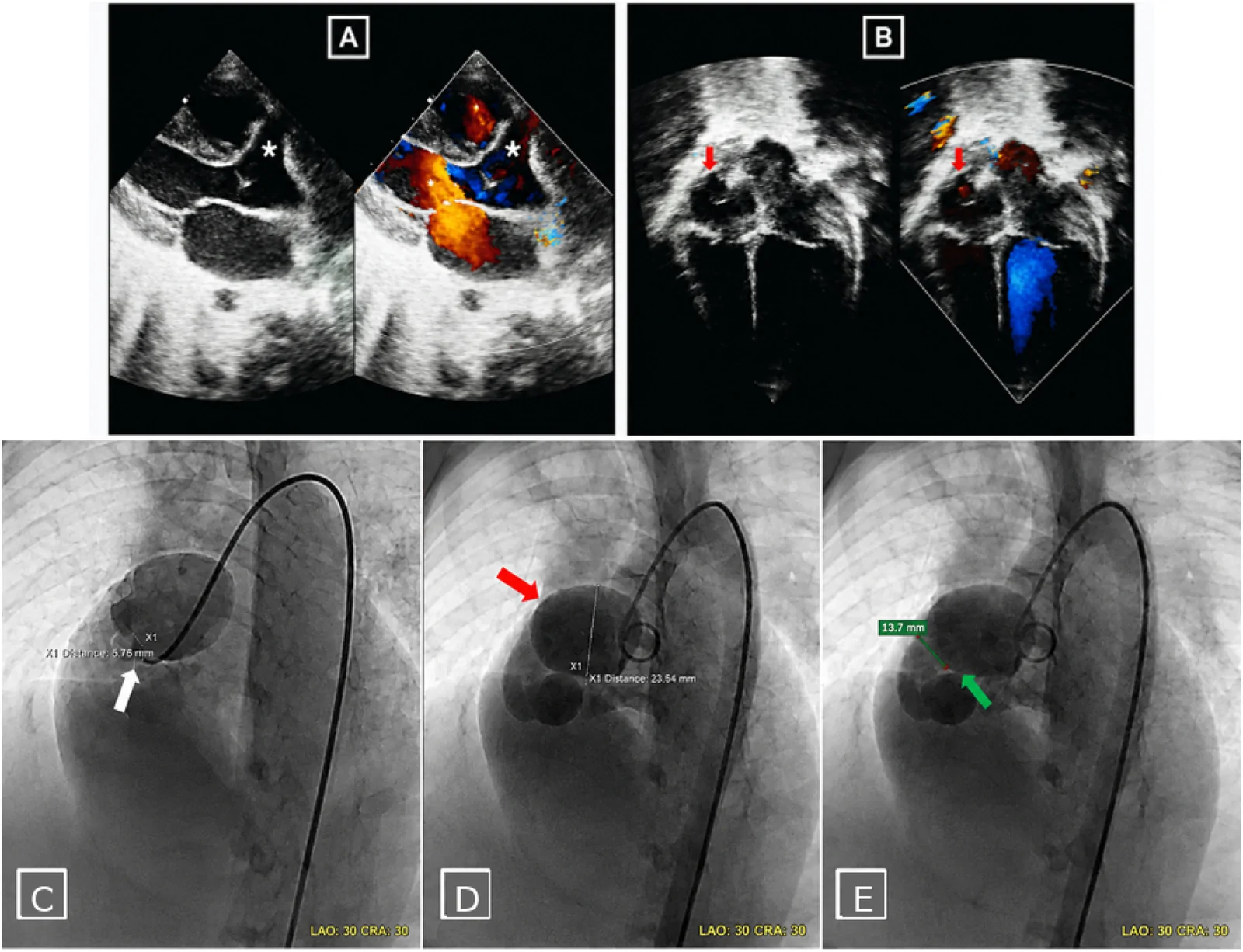
Transthoracic echocardiography demonstrating a markedly dilated right coronary artery (white asterisk) with a coronary artery fistula draining into right atrium (red arrow) in **(A)** Parasternal long-axis. **(B)** Apical four-chambers view with colour Doppler showing continuous fistulous flow into the right atrium. Selective right coronary angiography showing the angiographic anatomy of the coronary artery fistula **(C)** Dilatation of proximal RCA diameter measuring 5.76 mm (white arrow); **(D)** Giant aneurysmal sac (red arrow) arising immediately distal to dilated proximal RCA with maximal aneurysmal diameter measuring 23.54 mm (white arrow); **(E)** Tortuous fistulous tract beyond the aneurysm demonstrating multiple stenotic segments. The first more proximal stenotic segment, and immediately distal to the aneurysmal sac (green arrow) measuring 13.7 mm, was selected as the intended landing zone**.**

Cardiac catheterization was performed under general anaesthesia with endotracheal intubation. The peripheral baseline and arterial oxygen saturation was 99% with oxygen fraction (FiO_2_) 30%. Vascular access was obtained through 5-Fr sheaths in the right femoral vein (RFV) and right femoral artery (RFA). A 5-Fr pigtail catheter was advanced into the descending aorta followed by intravenous administration of heparin 50 IU/kg. Invasive hemodynamic measurements, intracardiac oxygen saturation measurements, saturation step-up assessment, and catheter-derived Qp/Qs calculations were not obtained. Ascending aortic angiography was subsequently performed to delineate the anatomy of the coronary fistula. The pigtail catheter was exchanged for a 5-Fr Judkins right (JR) diagnostic catheter.

Selective angiography performed through the JR diagnostic catheter demonstrated a markedly dilated proximal RCA measuring 5.76 mm (+10.72 z-score) giving rise to a giant tortuous coronary artery fistula draining into the right atrium. Immediately distal to the proximal RCA, the fistulous tract expanded into a large aneurysmal sac with a maximal diameter of 23.54 mm. Beyond the aneurysm, the fistulous tract followed a tortuous course with multiple sequential stenotic segments before distal draining into the right atrium. The first, more proximal stenotic segment, located immediately distal to the aneurysmal sac, measured approximately 13.7 mm and was selected as the intended landing zone for device deployment. It provided adequate separation from the native coronary branches while allowing stable anchoring of the occlusion device (Figures 2C–E). Based on the selected landing-zone diameter, a 20/16-mm Amplatzer® Vascular Plug II (AVP II) was selected for transcatheter closure via a transvenous approach using an arteriovenous loop. This device size was chosen in recommendation of approximately 30%–50% oversizing relative to the target vessel diameter (5).

Using a 0.032, 260-cm Terumo curved wire, the catheter was advanced into the RCA. The wire was successfully crossed through the fistulous tract into the RA, RV, and left pulmonary artery (LPA). A 5-Fr MPA2 catheter was subsequently advanced through the RFV into inferior vena cava (IVC), RA, RV, and LPA. A 10-mm snare was introduced through the MPA2 catheter into the LPA to capture the distal end of Terumo wire, thereby creating an arteriovenous loop.

The 5-Fr venous sheath was exchanged for an 8-Fr delivery sheath. The AVP II device was advanced through the delivery sheath from the right femoral vein toward the distal RCA. During the initial deployment under the LAO 30°/CRA 30° projection, the distal disc was positioned within the aneurysmal segment of the fistula and gently retracted to achieve stability, followed by gradual deployment of the proximal disc. However, repeated angiographic assessment demonstrated that this projection did not adequately delineate the intended landing zone because of overlap between the aneurysmal sac and the tortuous fistulous tract, making accurate assessment of device position difficult before the device was released, the device became unstable and was displaced toward the right atrium and inferior vena cava while remaining attached to the delivery cable (Figure 3). The device was fully recaptured into the 8-Fr delivery sheath, and the entire device–delivery system was withdrawn through the right femoral venous access. No snare or separate retrieval device was required. Following complete retrieval of the device and delivery system, new right venous punctures were performed, and the arteriovenous rail was re-established from the beginning for the second deployment attempt.

**Figure 3:**
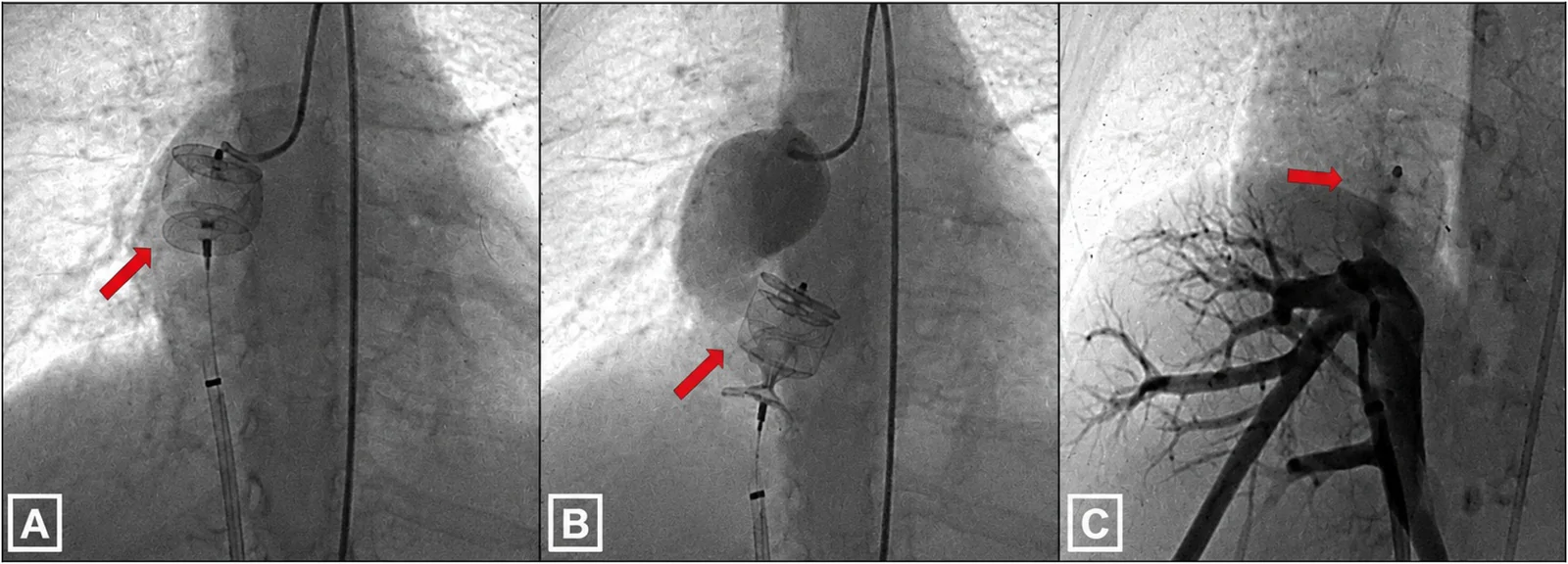
Failure of initial deployment resulting from suboptimal angiographic projection **(A)** Initial deployment of the Amplatzer® Vascular Plug II (red arrow) through the intended landing zone under the initial angiographic projection (LAO 30^0^/CRA 30^0^) with suboptimal device stability; **(B)** Device migration into right atrium following attempted deployment, indicating inadequate anchoring within fistulous tract; **(C)** Venous contrast injection demonstrating further migration of the unreleased device into the inferior vena cava, necessitating device recapture and subsequent redeployment.

The fluoroscopic projection was subsequently changed to LAO 50°/CAU 30°, which provided a more perpendicular visualization of the landing zone and better delineated the fistulous anatomy. To enhance device stability and preserve access across the tortuous fistulous tract in case retrieval or repositioning became necessary, the guidewire was intentionally maintained within the same 8-Fr delivery sheath, alongside the device delivery cable (Figure 4). Simultaneous contrast injection through the delivery sheath confirmed appropriate positioning of the device across the selected landing zone. Final angiography demonstrated stable device position, complete occlusion of the fistulous tract without residual shunt, and preserved native coronary artery flow. The activated clotting time was maintained between 200 and 250 s during procedure. The total volume of undiluted iodinated contrast medium administered was 180 mL (15.5 mL/kg). The total fluoroscopy time was 17 min 25 s, with a recorded cumulative radiation dose of 60 mGy.

**Figure 4:**
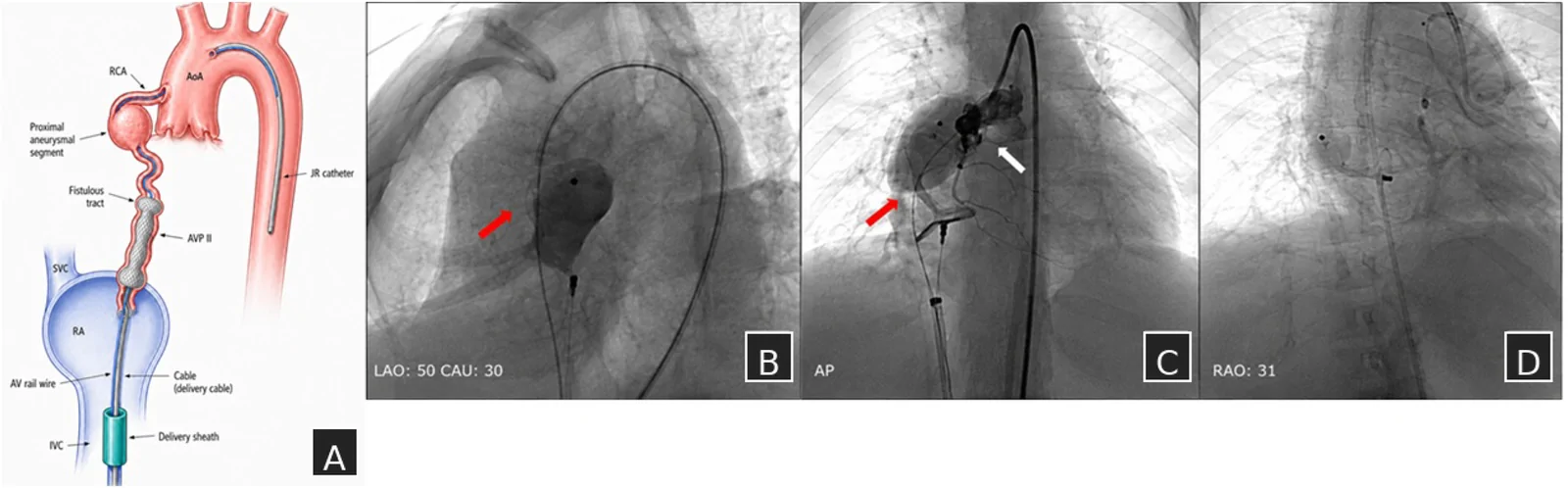
Successful redeployment using an optimized angiographic projection and maintained arteriovenous rail. **(A)** Schematic illustration of the wire–sheath–device relationship. The AV guidewire was maintained within the same delivery sheath alongside the device delivery cable; **(B)** Redeployment of the Amplatzer® Vascular Plug II (red arrow) under the optimized angiographic projection (LAO 50°/CAU 30°) with a maintained arteriovenous rail, providing improved visualization of the intended landing zone and enhanced device stability; **(C)** Selective hand injection through the JR diagnostic catheter confirming appropriate device position while preserving flow to the native right coronary artery branches (white arrow) before device release; **(D)** Completion angiography after device release demonstrating stable device position, preserved native coronary artery flow, and complete fistula occlusion.

Post-procedural chest radiography confirmed satisfactory device position over the right atrial region. The patient remained hemodynamically stable and was discharged on postoperative day 2 with aspirin (3–5 mg/kg/day) prescribed for 6 months. Follow-up transthoracic echocardiography demonstrated stable device position with complete occlusion of the fistula and no residual shunt. A 12-lead electrocardiogram obtained on the first postprocedural day showed no electrocardiographic evidence of myocardial ischemia or conduction abnormalities. At 1-month follow-up, the patient remained asymptomatic, with stable device position and complete fistula occlusion. The electrocardiogram, quantitative measurements of the proximal RCA/aneurysmal segment and right atrial and right ventricular dimensions were not recorded at this follow-up and therefore could not be reported.

## Discussion

Coronary artery fistulas (CAFs) are rare congenital coronary anomalies characterized by an abnormal communication between a coronary artery and a cardiac chamber or great vessels, bypassing the myocardial capillary bed. Approximately 50%–60% of the coronary artery fistulas originate from the RCA and most terminate in right-sided cardiac chambers because of lower-pressure receiving systems (1, 3).

The clinical manifestations depend primarily on fistula size and shunt magnitude. Small fistulas are frequently asymptomatic whereas large fistulas may cause significant left-to-right shunting, coronary steal phenomenon, progressive coronary artery dilatation, myocardial ischemia, arrhythmias, infective endocarditis, pulmonary over-circulation, and heart failure (1, 3, 6). In pediatric patients, recurrent respiratory infections and failure to thrive may represent early manifestations of chronic volume overload, as observed in our patient.

Transthoracic echocardiography remains the initial imaging modality for diagnosing CAF because it noninvasively demonstrates coronary artery dilatation, chamber enlargement, and abnormal fistulous flow. Nevertheless, selective coronary angiography remains the reference standard for defining complex fistulous anatomy and planning transcatheter closure, particularly in giant or tortuous CAFs (2, 3). Although cardiac computed tomography angiography provides excellent three-dimensional anatomical visualization, it was not performed in our patient because diagnostic catheter angiography had already been planned as part of the diagnostic and definitive management. Selective angiography provided real-time assessment of fistula morphology, coronary branch anatomy, landing-zone selection, device sizing, and procedural guidance (7). In our patient, angiography demonstrated a markedly dilated proximal RCA giving rise to a giant aneurysmal sac followed by a long tortuous fistulous tract with multiple sequential stenotic segments before draining into the right atrium. The proximal stenotic segment immediately distal to the aneurysm was selected as the intended landing zone because it provided stable device anchoring while preserving native coronary branches.

Various transcatheter occlusion devices have been described for CAF closure, including detachable coils, Amplatzer® vascular plugs, ductal occluders, and muscular ventricular septal defect occluders. Device selection should be individualized according to fistula morphology, vessel diameter, flow characteristics, and operator experience (3, 8). In the present case, an AVP II was selected because its multilayer nitinol mesh, symmetric three-lobed design, and controlled deployment characteristics provide secure anchoring within large high-flow vascular structures while reducing the risk of embolization compared with coil embolization (9, 10). These characteristics make AVP II particularly suitable for giant aneurysmal coronary artery fistulas.

A major technical challenge in this case was achieving stable device positioning within the aneurysmal and tortuous fistulous tract. Initial deployment under the conventional angiographic projection (LAO 30°/CRA 30°) was unsuccessful because its overlap between the aneurysmal sac and the tortuous fistulous tract obscured the intended landing zone, resulting in inadequate device apposition and subsequent migration into the right atrium and inferior vena cava. This experience highlights that successful transcatheter closure depends not only on appropriate device selection but also on accurate identification of the landing zone and optimal fluoroscopic projection. After changing the angiographic projection to LAO 50°/CAU 30°, the fistulous tract was visualized more perpendicularly, minimizing vessel overlap and allowing more accurate delineation of the landing zone before redeployment.

Another important technical aspect was maintaining the arteriovenous rail throughout redeployment. Preservation of the guidewire across the fistulous tract maintained continuous access, improved delivery sheath support, facilitated device retrieval after migration, enhanced coaxial alignment, and provided greater procedural stability during redeployment. To our knowledge, the procedural application of maintaining an AV rail throughout retrieval and redeployment has received little attention in interventional catheterization intervention for CAF or other congenital heart disease (11, 12). Our experience suggests that this simple bailout strategy may facilitate catheter support, improve device control during repeated repositioning, and increase procedural stability in giant, tortuous coronary artery fistulas with complex anatomy.

The goal of the treatment is to occlude the fistula to preserve the normal coronary blood flow. Identification of the number of fistulous communications, nature of the feeding vessels, the myocardium at risk and the sites of drainage help to optimize the procedural options available (13). Immediately before device release, selective hand injection through the JR diagnostic catheter confirmed preserved opacification of the native RCA branches, while simultaneous contrast injection through the delivery sheath verified accurate positioning of the device within the intended landing zone. This dual angiographic assessment increased procedural confidence before final device release.

Selection of the landing zone and occlusion device should be individualized according to fistula morphology rather than based solely on vessel diameter. In relatively short fistulas, proximal occlusion before an aneurysmal segment may successfully exclude the aneurysm while preserving native coronary flow (14). In contrast, our patient had a giant aneurysmal fistula followed by multiple sequential stenotic segments and marked tortuosity. Therefore, the first stenotic segment immediately distal to the aneurysm was considered the most suitable landing zone, providing adequate separation from native coronary branches and a more stable anchoring site. This strategy also allowed safe deployment of an appropriately oversized AVP II while minimizing the risk of device migration. Consistent with the design characteristics of the AVP II, device selection should be based on the intended landing-zone diameter rather than the maximal aneurysmal diameter. Oversizing relative to the landing zone improves frictional fixation and device stability, particularly in high-flow coronary artery fistulas, whereas sizing according to the aneurysmal sac may result in suboptimal anchoring (5).

Transcatheter closure has become the preferred treatment for anatomically suitable CAFs because it avoids sternotomy, shortens hospitalization, preserves native coronary circulation, and provides excellent procedural outcomes (2, 3). Although giant aneurysmal CAFs remain technically demanding because of their complex anatomy and increased risk of device instability, our case demonstrates that successful closure can be achieved through meticulous anatomical assessment, careful landing-zone selection, optimization of angiographic projection, and maintained arteriovenous rail-assisted deployment. These technical considerations may provide practical guidance for interventional pediatric cardiologists managing similarly complex coronary artery fistulas.

Despite successful immediate closure, patients with giant coronary artery fistulas require long-term surveillance because late complications may still occur. These include persistent aneurysmal dilatation, coronary thrombosis, residual shunting or recanalization, device embolization, myocardial ischemia related to altered coronary flow, and progressive coronary artery remodelling. Therefore, serial clinical evaluation, electrocardiography, echocardiography, and when clinically indicated, advanced coronary imaging is recommended after transcatheter closure, particularly in patients with giant aneurysmal fistulas (15).

## Limitations and literature context

This report is limited by its nature as a single-case experience with short-term follow-up; therefore, the generalizability of the procedural strategy cannot be established. In addition, multidetector computed tomography (MDCT), which can provide comprehensive three-dimensional anatomical assessment of coronary artery fistulas, was not performed. In our institution, however, the waiting time for pediatric cardiac MDCT is approximately 1–3 months, while diagnostic catheter angiography had already been planned because the patient had developed clinically significant left-to-right shunting with recurrent respiratory infections and poor weight gain requiring timely intervention. Consequently, catheter angiography was considered sufficient for both anatomical delineation and procedural planning without delaying treatment.

An additional procedural limitation was the relatively high volume of iodinated contrast medium administered (180 mL; 15.5 mL/kg). This was primarily attributable to the complex and severely tortuous fistula anatomy, the need for multiple angiographic projections, the unsuccessful initial device deployment, and repeated selective angiography to confirm device position, fistula occlusion, and preservation of the right coronary artery branches. Therefore, careful contrast minimization and monitoring of renal function should be considered in similar pediatric interventions. Long-term follow-up beyond 1 month was unavailable because the patient continued care with a pediatric cardiologist in her local region. Therefore, subsequent changes in fistula dimensions, right-sided chamber size, device-related thrombus, arrhythmia, and late myocardial ischemia could not be assessed.

Despite these limitations, this case contributes valuable procedural insights to the limited literature on transcatheter closure of giant tortuous right coronary artery-to-right atrium fistulas. Beyond demonstrating successful closure using an AVP II, this report emphasizes several practical technical considerations, including careful identification of the optimal landing zone, optimization of angiographic projection to minimize vessel overlap, maintenance of an arteriovenous rail during device retrieval and redeployment, and dual angiographic confirmation before final device release. These technical strategies may facilitate successful transcatheter closure in anatomically complex coronary artery fistulas and may serve as practical guidance for interventional pediatric cardiologists managing similar cases.

## Conclusions

Transcatheter closure of a giant and tortuous coronary artery fistula is achievable with meticulous anatomical assessment and thoughtful procedural planning. Optimization of angiographic projection, accurate landing zone selection, and maintenance of an arteriovenous rail may improve procedural stability and facilitate successful device deployment in anatomically complex fistulas. These procedural strategies may provide practical guidance for transcatheter closure of anatomically complex coronary artery fistulas in pediatric patients.

## Patient perspective

During follow-up visits, the patient's parents expressed satisfaction with the procedural outcome and reported that the child had resumed normal daily activities with improved exercise tolerance. No concerns regarding the procedure or follow-up care were raised.

## Data Availability

The original contributions presented in the study are included in the article/Supplementary Material, further inquiries can be directed to the corresponding author.

## References

[1] Qureshi SA. Coronary arterial fistulas. Orphanet J Rare Dis. (2006) 51:1–6. 10.1186/1750-1172-1-51 PubMed PMID: 17184545. PMC1764722 17184545

[2] Said SAM Van Der Werf T. Dutch Survey of coronary artery fistulas in adults: congenital solitary fistulas. Int J Cardiol. (2006) 106:323–32. 10.1016/j.ijcard.2005.01.047 PubMed PMID: 16337040. 16337040

[3] Armsby LR Keane JF Sherwood MC Forbess JM Perry SB Lock JE. Management of coronary artery fistulae: patient selection and results of transcatheter closure. J Am Coll Cardiol. (2002) 39:1026–32. 10.1016/S0735-1097(02)01742-4 PubMed PMID: 11897446. 11897446

[4] Gowda RM Vasavada BC Khan IA. Coronary artery fistulas: clinical and therapeutic considerations. Int J Cardiol. (2006) 107:7–10. 10.1016/j.ijcard.2005.01.067 PubMed PMID: 16125261. 16125261

[5] Ramakrishnan S. Vascular plugs - A key companion to interventionists - “just ppug it.”. IHJ. (2015) 67:399–405. 10.1016/j.ihj.2015.07.001 PubMed PMID: 26304581. PMC4561770 26304581

[6] Latson LA. Coronary artery fistulas: how to manage them. Catheter Cardiovasc Interv. (2007) 70:110–6. 10.1002/ccd.21125 PubMed PMID: 17420995. 17420995

[7] Li YF Zhang ZW Wang SS Xie ZF Zhang X Li YF. Transcatheter closure of congenital coronary artery fistulas with a giant coronary artery aneurysm in children: experiences from a single center. Chin Med J (Engl). (2017) 130:1919–25. 10.4103/0366-6999.211894 PubMed PMID: 28776543. 28776543 PMC5555125

[8] Xu L Xu ZY Jiang SL Zheng H Zhao SH Ling J. Transcatheter closure of coronary artery fistula in children. Chin Med J (Engl). (2010) 123:822–6. 10.3760/cma.j.issn.0366-6999.2010.07.011 PubMed PMID: 20497671. 20497671

[9] Mangukia CV. Coronary artery fistula. Ann Thorac Surg. (2012) 93:2084–92. 10.1016/j.athoracsur.2012.01.114 PubMed PMID: 22560322. 22560322

[10] Mcmahon CJ Nihill MR Kovalchin JP Mullins CE Grifka R. Coronary artery fistula: management and intermediate-term outcome after transcatheter coli occlusion. Tex Heart Inst J. (2001) 28:21–5. 11330735 PMC101124

[11] Zampi JD Whiteside W. Innovative interventional catheterization techniques for congenital heart disease. Translational Pediatr. (2018) 7:104–19. 10.21037/tp.2017.12.02 PMC5938250 29770292

[12] Trang P-TT Cuong TC Cuong NM Tin DN Tran Tran N Thang LM. Giant coronary artery fistula: a case report. Radiol Case Rep. (2021) 18:2621–7. 10.1016/j.radcr.2023.05.003 PMC10238605 37273728

[13] Yadav A Buxi TBS Jayamma SS Rawat KS Ghuman SS Agarwal N. Coronary cameral fistula treated by the amplatzer® vascular plug. Jpn J Radiol. (2014) 32:608–12. 10.1007/s11604-014-0335-8 PubMed PMID: 24854902. 24854902

[14] Jang HI Choi YE Cho HJ Cho YK Ma JS. Transvenous proximal closure of large congenital coronary arteriovenous fistula using the single amplatzer® vascular plug in a 3-year-old girl. Korean J Pediatr. (2013) 56:90–3. 10.3345/kjp.2013.56.2.90 23482898 PMC3589597

[15] Al-Hijji M El Sabbagh A El Hajj S AlKhouli M El Sabawi B Cabalka A. Coronary artery fistulas: indications, techniques, outcomes, and complications of transcatheter fistula closure. JACC: Cardiovasc Interv. (2021) 14:1393–406. 10.1016/j.jcin.2021.02.044 PubMed PMID: 34238550. 34238550

